# Polymorphic Variants of Peptidylarginine Deiminase Gene from *P. gingivalis*—Searching for Targets for Supportive Therapy of Periodontitis

**DOI:** 10.3390/ijms26041662

**Published:** 2025-02-15

**Authors:** Karolina Strzelec, Agata Dziedzic-Kowalska, Łukasz Sieron, Grzegorz P. Bereta, Karolina L. Stepien, Klara Ferenc, Katarzyna Łazarz-Bartyzel, Iwona Olszewska-Czyż, Iwona Rąpalska, Małgorzata Aptekorz, Tomasz Kaczmarzyk, Marta Cześnikiewicz-Guzik, Katarzyna Gawron

**Affiliations:** 1 Department of Molecular Biology, Faculty of Medical Sciences in Katowice, Medical University of Silesia, 40-752 Katowice, Poland; karolina.strzelec@sum.edu.pl (K.S.); agata.dziedzic@sum.edu.pl (A.D.-K.); kbugaj@sum.edu.pl (K.L.S.); s87943@365.sum.edu.pl (K.F.); 2 Department of Medical Genetics, Faculty of Medical Sciences in Katowice, Medical University of Silesia, 40-752 Katowice, Poland; lukasz.sieron@sum.edu.pl; 3 Malopolska Centre of Biotechnology, Jagiellonian University, 30-387 Krakow, Poland; grzegorz.bereta@uj.edu.pl; 4 Department of Periodontology, Preventive Dentistry and Oral Pathology, Faculty of Medicine, Medical College, Jagiellonian University, 31-155 Krakow, Poland; katarzyna.lazarz-bartyzel@uj.edu.pl (K.Ł.-B.); iwona.olszewska-czyz@uj.edu.pl (I.O.-C.); marta.czesnikiewicz-guzik@uj.edu.pl (M.C.-G.); 5 Department of Oral Surgery, Medical College, Jagiellonian University, 31-155 Krakow, Poland; iwona.rzasa@uj.edu.pl (I.R.);; 6 Department of Medical Microbiology, Faculty of Medical Sciences in Katowice, Medical University of Silesia, 40-752 Katowice, Poland; maptekorz@sum.edu.pl

**Keywords:** periodontitis, *P. gingivalis*, virulence factors, peptidylarginine deiminase, gene sequence, gene variability, pro-inflammatory cytokines, PGE_2_-dependent pathway

## Abstract

Periodontitis (PD), an oral inflammatory disease, is primarily caused by *P. gingivalis*. Peptidylarginine deiminase (PPAD) is considered an attractive virulence factor because, due to protein citrullination, it may have deleterious effects on host tissues. In this study, the *ppad* gene sequences from *P. gingivalis* were analyzed in the context of its impact on bacterial virulence and potential targets for PD therapy. Analyses of *ppad* sequences from 58 patients with various clinical stages of PD, 20 controls, and 60 sequences from public databases were conducted. Overall, 55 substitutions assigned as polymorphic variants (4), missense mutations (10), or synonymous variants (35) were identified in PD, and 22 synonymous variants were identified in controls. Among them, the G231N, E232T, N235D variant was found in ~25% of *P. gingivalis* strains from PD samples. It was located close to the catalytic triad and had two-fold higher activity in comparison with reference *P. gingivalis,* upregulated expression of key inflammatory mediators, and contributed to worsening periodontium conditions in advanced PD, suggesting their unambiguous impact on *P. gingivalis* virulence. Our results indicate the G231N, E232T, N235D variant of the *ppad* gene as a potential candidate, opening a path to searching for novel targets for supportive therapy of PD. Further validation of the identified mutations is needed in future studies.

## 1. Introduction

Periodontitis (PD) is the most common inflammatory disease of the oral cavity caused by bacterial infections. According to epidemiological data, it affects about 50% of adults worldwide [1,2]. The proliferation of specific Gram-negative bacteria in subgingival plaque causes chronic inflammation of tooth-supporting structures, destruction of the periodontal ligament, and resorption of alveolar bone, and untreated, it may lead to tooth loss. One of the major etiological factors of this disease is an anaerobic, non-motile, Gram-negative bacterium, *P. gingivalis* [3,4,5,6,7]. To date, a variety of virulence factors, including lipopolysaccharides, proteinases, and hemagglutinins, have been broadly investigated, and their contribution to bacterial pathogenicity is evident [8,9,10,11,12].

In recent years, there has been increasing interest in peptidylarginine deiminase (PPAD), a non-proteolytic enzyme expressed by *P. gingivalis*. PPAD catalyzes the citrullination of arginine in proteins and peptides, with a preference for C-terminal Arg residues, to produce peptidylcitrulline and ammonia. The citrullination of free arginine provides energy for anaerobic growth, while the ammonia generated by this system enables *P. gingivalis* survival during acid-cleansing cycles of the oral cavity [13,14,15,16,17]. PPAD belongs to the guanidino-modifying enzyme superfamily (GME) and is secreted by the type IX secretion system (T9SS), also referred to as the Por secretion system (PorSS) [18,19]. PPAD is characterized by the presence of the following structural elements: N-terminal signal peptide (NtSP), catalytic domain (CD), immunoglobulin-like fold domain (IgLF), and C-terminal domain (CTD). The CD domain contains the active site of the enzyme and is responsible for the full biological activity of PPAD [18]. PPAD, located in the outer membrane of *P. gingivalis*, allows direct modification of its cell envelope proteins and host proteins [20]. Citrullination catalyzed by PPAD decreases the charge of modified proteins; thus, it may also affect protein stability, sensitivity to proteolysis, and biological activity. For instance, Pyrc et al. reported that PPAD efficiently citrullinates the carboxy-terminal arginine of epidermal growth factor (EGF), and this modification subsequently impairs the biological function of this cytokine, which may contribute, at least partially, to tissue damage and delayed healing in PD [21]. Moreover, our previous study showed that PPAD contributes to the effective adhesion and invasion of primary human gingival fibroblasts (PHGFs) by *P. gingivalis* and to the significant upregulation of the prostaglandin E_2_ (PGE_2_)-dependent pathway during infection of fibroblasts by this pathogen [22]. We also found that PPAD knockout strain hindered the pathogen’s ability to adhere to and invade PHGFs. Several reports indicated that protein citrullination catalyzed by PPAD may contribute to the pathogenesis of PD and rheumatoid arthritis (RA) [23,24]; for the latter, citrullination of bacterial and host proteins by PPAD in inflamed gingival tissues is considered a molecular mechanism for generating antigens that initiate and/or enhance the autoimmune response in RA [20,24,25,26,27,28]. *P. gingivalis* pathogenicity varies depending on the strains, whereas PPAD is considered an attractive virulence factor of this bacterium, hence, in the current study, we endeavoured to identify mutations in the *ppad* gene of *P. gingivalis* in the context of their impact on bacterial virulence and potential targets to support PD therapy.

## 2. Results

### 2.1. Verification of the Genotypes of P. gingivalis Clinical Strains

**Figure 1:**
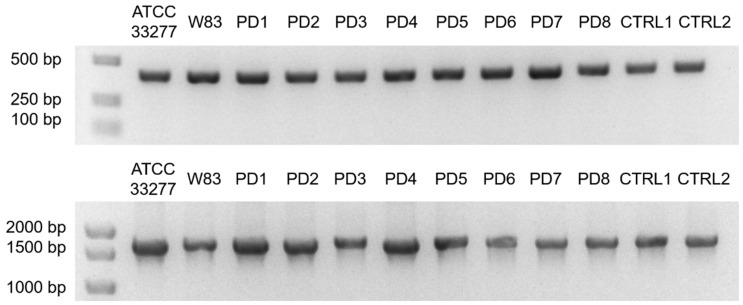
Agarose gel electrophoresis of PCR products of *16S rRNA* (**upper panel**) and *ppad* (**lower panel**) genes specific for *P. gingivalis*. Representative clinical strains from PD patients and control donors are presented. W83 and ATCC 33277, laboratory reference *P. gingivalis* strains; PD, periodontitis; PD1-2, mild; PD3-4, moderate; PD5-6, moderate/advanced; PD7-8, advanced; CTRL1-2, healthy donors.

GCF samples were collected from 138 donors diagnosed with PD and 69 control healthy donors. Homogeneous strains of *P. gingivalis* were obtained from 58 PD donors and 20 controls. Genomic DNA isolation was conducted to confirm the presence of *P. gingivalis* species using PCR specific for *16S rRNA* and the *ppad* gene. Representative clinical strains from PD patients and control donors are presented in Figure 1.

### 2.2. Clinical Classification of PD in Study Donors

PD diagnosis was confirmed on the basis of periodontal examination, which also included medical history and X-rays. The control donors were characterized by the values of clinical parameters within the reference range. The study group was classified as mild, moderate, moderate/advanced, and advanced PD. The number, range of age, and gender of PD and control donors are presented (Table 1).

### 2.3. Overview of ppad Gene Sequences from P. gingivalis Clinical Strains

Analysis of *ppad* nucleotide sequences from PD-derived *P. gingivalis* strains showed significant diversity in comparison with the reference *P. gingivalis* ATCC 33277 (wild-type) strain. Overall, in *ppad* gene sequences from PD-derived *P. gingivalis* strains, fifty-five nucleotide substitutions were identified, which were assigned as polymorphic variants, missense mutations, or synonymous variants, respectively. A total of 20 substitutions (36.36%) resulted in modification of the encoded protein sequence, whereas 35 (63.64%) were classified as synonymous variants. In total, 4 polymorphic variants and 10 missense mutations were identified. In the control group, 22 nucleotide substitutions were detected, of which 22 (100.00%) were synonymous variants (Table 2). The changes are located throughout the entire protein-coding sequence, without particular emphasis on any part of PPAD.

### 2.4. Identification of New Specific Polymorphic Variants in P. gingivalis ppad Gene from PD, But Not Control Donors

**Figure 2:**
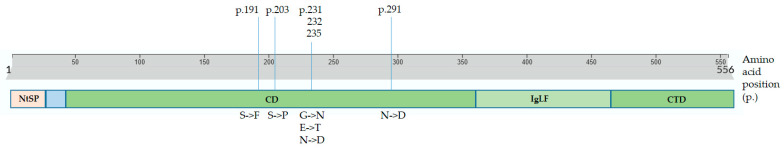
Schematic of the whole PPAD amino acid sequence (1–556) with identified polymorphic variants (S191F; S203P; G231N, E232T, N235D; N291D). The region marked in red is the N-terminal signal peptide (NtSP), blue—propeptide, green—PPAD chain containing catalytic domain (CD), immunoglobulin-like folded domain (IgLF), and C-terminal domain (CTD). The “p.” means amino acid position in the PPAD sequence. The figure was made using Uniprot databases.

In total, fifty-five nucleotide substitutions were identified in the PD group, amongst which a total of 49 changes were identified in the *ppad* sequences from advanced PD. Notably, those patients were infected by *P. gingivalis* strains harboring 4 (8.16%) novel polymorphic variants. In brief, the S191F variant was found in 12; S203P in 10; G231N, E232T, N235D in 11; and N291D in 16 of 37 donors with advanced PD, respectively. Most substitutions resulting in a change of the encoded protein in the advanced PD did not occur in the other groups of PD donors, i.e., mild, moderate, and moderate/advanced groups. An exception was the S191F and N291D variants. The N291D substitution was observed in moderate and moderate/advanced PD and co-existed with the S191F variant in moderate and advanced PD. Additionally, the S191F variant was present in mild, moderate, moderate/advanced, and advanced PD. The transition of a single nucleotide was identified in 8 of 10 substitutions, whereas the transversion was less frequent. Conversely, we did not identify any polymorphic variants in the control group. In silico analysis also did not allow us to observe any change in the chemical nature of amino acids due to specific substitutions. Taking together, among 49 changes found in the *ppad* sequences from the patients diagnosed with advanced PD, 4 new polymorphic variants were detected. The mild and moderate PD was characterized by the presence of the same two polymorphic variants (S191F, N291D), while in the moderate/advanced group, all four variants were found to occur (Figure 2, Table 3 and Table 4).

### 2.5. Missense Mutations Detection and Co-Occurrence with New Polymorphic Variants of the P. Gingivalis ppad Gene from PD

In total, 10 missense mutations were identified in the *P. gingivalis ppad* gene sequence obtained from the PD group, and each mutation occurred as a separate change within the analyzed gene sequence. The most frequent missense mutation in the PD group was A515V, which also co-occurred with the S203P and G231N, E232T, N235D polymorphic variant in the *ppad* sequences from *P. gingivalis* strains isolated from advanced PD (10/37). The second most common substitution characteristic of advanced PD was S528G. Other mutations were T275P and P536L, which were detected exclusively in *P. gingivalis* strains isolated from advanced PD. Next, the M77V and Q373K mutations were identified within the *ppad* gene sequences from *P. gingivalis* strains isolated from moderate and advanced PD patients, and the latter co-existed with the S191F polymorphic variant. Beyond that, we did not detect any specific missense mutation in *P. gingivalis* strains derived from mild, moderate, and moderate/advanced PD. Summarizing, among 10 missense mutations identified in PD group, the most attractive candidates for further studies seem to be A515V and S528G, being the most commonly present in PD and co-existing with S203P and the G231N, E232T, N235D polymorphic variant in advanced PD, and T275P and P536L, which occurred exclusively in advanced PD (Table 5).

### 2.6. Identification of Numerous Synonymous Variants of the P. gingivalis ppad Sequence from PD and Controls

In brief, 35 synonymous variants were identified in PD, and 22 were identified in controls. The most frequent substitution was the transition of cytosine with thymine, while the transversion occurred sporadically. The most common synonymous variants of the *P. gingivalis ppad* sequence from the PD were T200T and V284V, while the frequency of individual changes in healthy donors did not differ from each other. Interestingly, a synonymous variant characteristic for three PD groups was detected. In brief, in mild PD, no characteristic variants were observed. In the moderate group, the L264L variant (1.72%) was distinguished, while in moderate/advanced PD, Y126Y was distinguished (3.45%). In advanced PD, S206S (12.07%) and P457P (8.62%) variants were detected.

In advanced PD infected by *P. gingivalis* producing the G231N, E232T, N235D variant of the *ppad* gene, three synonymous variants were identified at the 121st, 206th, and 457th amino acid residues, respectively. Of note, these changes were not present in the nucleotide sequence of the *ppad* gene from remaining patients, i.e., infected by *P. gingivalis* strains without the 7-nucleotide polymorphism of the *ppad* and control donors. Moreover, the substitutions contributing to the synonymous variants in the majority referred to hydrophobic amino acids (22/35 PD group and 13/22 controls). These results showed that the synonymous variants represent the largest number of nucleotide substitutions characterized by meaningful diversity in both PD and healthy groups (Table 6).

### 2.7. Characteristics of the P. gingivalis ppad Sequence Changes Located Close to the Active Center of PPAD

Based on in silico secondary structure analysis, 11 changes corresponding to 130th, 236th, 238th, 297th, and 351st conserved amino acid residues known to be involved in catalysis of PPAD were distinguished [18,29]. Changes located within 10 amino acids from the active site residues were in close proximity to PPAD.

Changes in four codons (14.54%) were classified as polymorphic variants (G231N, E232T, N235D, and N291D) in the PD group, while seven substitutions (12.72%), i.e., Y126Y, F135F, A245A, N247N, R304R, G345G, and G360G, were designated as synonymous variants. Moreover, the occurrence of nucleotide substitutions in close proximity to the active site was mostly related to hydrophilic amino acids.

Summarizing, close proximity to the active center of PPAD revealed diversity in the clinical strains of *P. gingivalis*, which may result in diverse enzymatic activity. Presentation of the specific changes of the *P. gingivalis ppad* sequence located in close proximity to the active site of PPAD is shown in Table 7.

### 2.8. Analysis of ppad Gene Sequences Deposited in Databases

An analysis of the *ppad* gene sequences of *P. gingivalis* was conducted using sequences deposited in publicly available databases, specifically GenBank and NCBI. A total of 60 sequences corresponding to the *ppad* gene from various *P. gingivalis* strains were identified. These sequences covered the complete coding sequence of the *ppad* gene (556 amino acids) to assess nucleotide substitutions. Sequences containing deletions, insertions, or those annotated as originating from clinical strains isolated from patients with systemic diseases, such as RA, were excluded from the analysis.

The analysis identified a total of 104 nucleotide substitutions, which were categorized into polymorphic variants, missense mutations, or synonymous variants. The in silico analysis confirmed substantial diversity in the coding sequences of the *ppad* gene.

Three polymorphic changes were identified within the analyzed dataset: S203P (30% frequency); G231N, E232T, N235D (25% each); and N291D (40%) (Table S3). These variants and their frequencies align with the results obtained in our clinical dataset. Notably, the S191F variant, which exhibited a frequency of 20%, was absent among the polymorphic variants in database-derived sequences and was therefore assigned to the missense mutation group.

The group of missense mutations exhibited significantly higher diversity. A total of 36 missense mutations were identified (Table S4), and their frequency ranged between 1.67% and 20.00%. The most frequent substitutions were S191F (20%) and M77V (18.33%), both of which were also detected in our clinical sequences. Additionally, five mutations resulted from more than one nucleotide substitution in the *ppad* gene: A96Q (1.67%), S95N (1.67%), A121T (1.67%), K122Y (1.67%), and A136I (1.67%). Interestingly, multi-nucleotide substitutions were exclusively observed among polymorphic variants in our clinical dataset. Single-nucleotide transitions were also more common than transversions in this group.

Synonymous variants formed the largest group, with 53 distinct changes identified (Table S5). Each synonymous variant resulted from a single-nucleotide substitution, and their frequency ranged between 1.67% and 98.33%. The most frequent synonymous variants were V531V (98.33%), Y71Y (63.33%), and I497I (63.33%), which also aligned with the results obtained in our clinical dataset. Other synonymous variants present in database sequences but absent in our study exhibited lower frequencies.

The current analysis revealed 16 substitutions located in close proximity to the active site of PPAD. These included two polymorphic variants (G231N, E232T, N235D; N291D), six missense mutations (A121T, K122Y, A132A, A136L, P294L, and A357V), and eight synonymous variants (A121A, T123T, Y126Y, F135F, A245A, R304R, G345G, and G360G). The presence of nucleotide substitutions near the active site was predominantly associated with hydrophilic amino acids.

The results of the *ppad* gene sequence analysis from database-derived sequences unequivocally confirmed the significant genetic heterogeneity of the analyzed gene. However, due to the lack of available clinical data for these sequences, it was not possible to fully correlate our findings with the described variants. Consequently, we cannot determine whether the observed changes predispose to the development of more advanced clinical forms of periodontal disease.

### 2.9. In Vitro Analysis of Immune Response by PHGFs Infected with P. gingivalis Strains Harboring the G231N, E232T, N235D Polymorphic Variant of ppad

**Figure 3:**
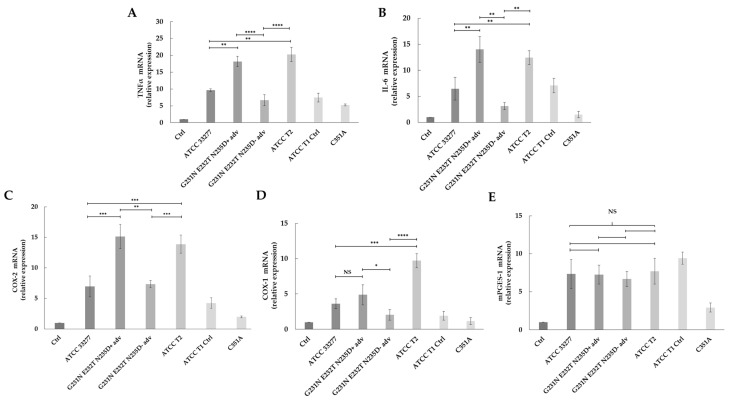
Analysis of immune response by cells infected with *P. gingivalis* strains producing the G231N, E232T, N235D polymorphic variant of *ppad*. Relative expression levels of tumor necrosis factor α (*TNF-α*) (**A**), interleukin-6 (*IL-6*) (**B**), cyclooxygenase-2 (*COX-2*) (**C**), cyclooxygenase-1 (*COX-1*) (**D**), and microsomal PGE synthase-1 (*mPGES-1*) (**E**) in primary human gingival fibroblasts (PHGFs) obtained from two PD donors and infected for 24 h at MOI of 100 with reference wt-*P. gingivalis* strain (ATCC 33277); clinical *P. gingivalis* strain harboring the G231N, E232T, N235D variant isolated from advanced PD donor (G231N, E232T, N235D+ adv); *P. gingivalis* strain without polymorphic variant obtained from a donor with advanced PD (G231N, E232T, N235D- adv); *P. gingivalis* ATCC 33277 strain modified to code the G231N, E232T, N235D variant (ATCC T2); and its control strain with only antibiotic resistance cassette introduced in *P. gingivalis* ATCC 33277 (ATCC T1 Ctrl), a control ATCC 33277 strain, which produces a catalytically inactive form of PPAD (C351A); uninfected cells (Ctrl). Quantitative real-time PCR was performed using *β-actin* as a reference gene. Data represent four independent experiments and are expressed as means ± SD (**** *p* < 0.0001; *** *p* < 0.001; ** *p* < 0.01; * *p* < 0.05; and NS—non significant statistically).

We recently reported that the severity of PD correlated with a higher level of PPAD activity that was associated with the presence of a triple mutation (G231N, E232T, N235D) in PPAD in comparison with W83 and ATCC 33277-type strains [30]. This polymorphic variant is located in the vicinity of the catalytic triad, close to His236 of PPAD, and it was found in ~25% of *P. gingivalis* strains isolated from PD patients [30], as confirmed in the current study. Therefore, we subsequently attempted to verify whether *P. gingivalis* strains harboring the G231N, E232T, N235D polymorphic variant of PPAD can affect an immune response through modulation of tumor necrosis factor α (TNF-α) and interleukin-6 (IL-6). Infection of gingival fibroblasts with a clinical *P. gingivalis* strain harboring the G231N, E232T, N235D variant isolated from advanced PD donor (G231N, E232T, N235D+ adv) or with a laboratory mutant of the G231N, E232T, N235D variant introduced to the ATCC 33277 *P. gingivalis* strain (ATCC T2) upregulated the expression of *TNF-α* (Figure 3A) and *IL-6* (Figure 3B). The expression of each gene was significantly elevated compared to control wt-*P. gingivalis* (ATCC 33277 strain) (*p* < 0.01). Remarkably, infection of fibroblasts with the use of either, G231N, E232T, N235D+ adv or the ATCC T2 *P. gingivalis* strain resulted in elevated expression of *TNF-α* (*p* < 0.0001) (Figure 3A) or *IL-6* (*p* < 0.01) (Figure 3B) in comparison with their expression by PHGFs infected with *P. gingivalis* strain without polymorphic variant obtained from a donor with advanced PD (G231N, E232T, N235D- adv). Moreover, upregulated expression of both genes was strongly reduced in cells infected with the C351A strain, which produces a catalytically inactive form of PPAD.

In the previous study, we demonstrated that PPAD activity is implicated as an important factor for gingival fibroblast infection and activation of PGE_2_ synthesis, the latter of which may strongly contribute to bone resorption and eventual tooth loss [22]. Moreover, the introduction of triple point mutations (G231N, E232T, N235D) in PPAD significantly increased its activity compared with their parental strains (ATCC 33277 and W83); the purified PPAD enzyme with all three mutations also showed higher enzymatic activity than the non-mutated enzyme [30]. Considering the crucial impact of the PGE_2_-dependent pathway in PD pathogenesis, we subsequently analyzed whether the G231N, E232T, N235D polymorphic variant may affect the expression of cyclooxygenase-1 (*COX-1*), cyclooxygenase-2 (*COX-2*), and microsomal PGE synthase-1 (*mPGES-1*).

As depicted in Figure 3C, the expression of COX-2 was upregulated in the case of PHGFs infected by either ATCC T2 or G231N, E232T, N235D+ adv *P. gingivalis* strains; infection using each of those bacterial strains resulted in significantly increased expression of *COX-2* compared to the wt ATCC 33277 strain (*p* < 0.001). *COX-2* expression following cells infection with the *P. gingivalis* strain without a polymorphic variant obtained from a donor with advanced PD (G231N, E232T, N235D- adv) was significantly reduced in comparison with its expression due to infection with the G231N, E232T, N235D+ adv *P. gingivalis* strain (*p* < 0.01) and infection with the ATCC T2 *P. gingivalis* strain (*p* < 0.001) (Figure 3C). Regarding the relative expression of *COX-1*, it was upregulated after infection of PHGFs by the ATCC T2 *P. gingivalis* strain, and it was significantly elevated in comparison with *COX-1* expression after infection with control wt-*P. gingivalis* (ATCC 33277 strain, *p* < 0.001) and infection with the *P. gingivalis* strain without the polymorphic variant obtained from a donor with advanced PD (G231N, E232T, N235D- adv, *p* < 0.0001). The expression of *COX-1* in PHGFs infected with clinical *P. gingivalis* strain harboring the G231N, E232T, N235D variant isolated from an advanced PD donor (G231N, E232T, N235D+ adv) was also upregulated in comparison with its expression level observed for cells infected with the clinical *P. gingivalis* strain without the G231N, E232T, N235D polymorphism from a donor with advanced PD (G231N, E232T, N235D- adv, *p* < 0.05), and compared with *COX-1* expression after infection with ATCC 33277, but in the latter case, the difference of expression was not statistically significant (*p* = 0.178) (Figure 3D).

Increased expression of *mPGES-1* was found following cell infection with each *P. gingivalis* strain, excluding the C351A mutant; we did not observe, however, any differences of *mPGES-1* expression due to infection with the bacterial strain expressing the G231N, E232T, N235D variant, a strain without this variant, or wt ATCC 33277 *P. gingivalis* (Figure 3E).

In summary, the current results demonstrate that gene expression in gingival fibroblasts is dynamically modulated by *P. gingivalis* strains harboring the G231N, E232T, N235D variant, driving the heightened immune response characteristic of PD.

## 3. Discussion

In recent years, an increasing number of studies have uncovered the significance of PPAD, its enzymatic activity, and possibly bacterial and/or host protein citrullination in the virulence of *P. gingivalis* [13,14,15,16,17]. For example, it has been proven that the functionality of EGF was significantly impaired as a result of effective citrullination, hindering wound healing in the course of PD [21]. In another study, Bielecka et al. analyzed the influence of bacterial citrullination on the pro-inflammatory properties of anaphylatoxin C5a and demonstrated reduced chemotactic properties of neutrophils as a result of the reaction catalyzed by PPAD [28]. Our previous study demonstrated that PPAD activity contributes to *P. gingivalis* adherence to and invasion of gingival fibroblasts, as well as PGE_2_-pathway activation in infected fibroblasts [22]. More recently, we identified a new super-active genetic variant of *ppad* produced by *P. gingivalis* strains colonizing advanced PD [30]. Although PPAD attracted an increasing interest as a main player in a number of studies, the association of mutated variants of the *ppad* gene with bacterial pathogenicity and their potential use as new genetic targets for PD therapy has not been discussed in the scientific literature.

In the current study, 55 nucleotide substitutions assigned as polymorphic variants, missense mutations, or synonymous variants were identified in the *ppad* gene sequences from PD-derived *P. gingivalis* strains, and 22 nucleotide substitutions in controls, of which all were classified as synonymous variants. Specifically, we identified 2 new polymorphic variants located in close proximity to the active site (i.e., within 10 amino acids from the active site residues) of the PPAD enzyme and 10 missense mutations in the PD group, which was opposite to the controls.

These data showed that the *ppad* gene sequence is characterized by considerable heterogeneity, particularly in the PD group. This observation was contrary to the study performed by Gabarrini et al., who did not detect in *ppad* gene substitutions inducing the modification of encoded protein sequence. Interestingly, no mutation within the signal peptide region or in the neighbourhood of the active center was identified [31]. The discrepancies between the data from our laboratory and Gabarrini’s may result from different methodologies used. In the current study, the whole *ppad* gene of *P. gingivalis* strains isolated from PD patients and healthy donors was sequenced. Subsequently, the complete PPAD protein coding sequences were analyzed against the reference sequence of the wild-type strain of *P. gingivalis* using the BLAST program. In contrast, Gabarrini et al. used the restriction enzyme digestion method, hence, they would not identify any changes in the *ppad* gene. Moreover, Gabarrini et al. performed analyses in 7 of the 92 clinical *P. gingivalis* strains which they collected, which may suggest that the number of analyzed samples was insufficient to fully verify potential mutations present in *ppad* gene sequences [31].

The first group of *ppad* gene changes we searched for were polymorphic variants. One of the studied variants of the *ppad* gene was the seven-nucleotide substitution responsible for a three amino acid G231N, E232T, N235D change in the primary structure of the enzyme. It was found in ~25% of *P. gingivalis* strains isolated from PD patients and located in the vicinity of the catalytic triad, close to His236, which corroborates with 2-fold higher enzymatic activity than that in the reference *P. gingivalis* ATCC 33277 [29,30]. Moreover, as reported in our recent study, the presence of this polymorphic variant correlated with worse clinical indices of disease severity, including the probing pocket depth (PPD) and clinical attachment level (CAL) in advanced PD [30]. Additionally, crystallographic models of the PPAD protein structure indicated the differences between the PPAD T1 and PPAD T2 (G231N, E232T, N235D), located in the loop, close to the active center of the PPAD. The E232T mutation involves the change of glutamic acid to neutral threonine and is located in the area essential to the deimination reaction. It forms the channel through which the water molecule passes and ammonia during citrullination. The substitution of glycine to asparagine (G231N) may also influence the conformation of this channel. Additionally, asparagine 235 is located in the channel through which the hydroxyl ion passes to restore the active center of the enzyme to a state ready to accept the substrate. Thus, the N235D mutation induces the change of asparagine to negatively charged aspartic acid. Therefore, the changes of amino acids of individual variants discussed herein can clearly explain the change of catalytic properties of the enzyme [18,19,30,32].

Another polymorphic variant identified close to the catalytic triad (residues Asn297) was the N291D substitution, which induces the change of asparagine to negatively charged aspartic acid. This variant was detected in ~35% of *P. gingivalis* strains from PD, but its association with a specific stage of PD was not observed. Being located near the active site, the change from asparagine to aspartic acid could alter the electrostatic environment, potentially affecting substrate binding or catalytic efficiency. Moreover, the introduction of a negative charge may disrupt existing hydrogen bonds or introduce new electrostatic interactions that could either stabilize or destabilize the active site [33,34]. On the other hand, the co-occurrence of the N291D with the S191F substitution was associated with moderate and advanced PD. Since the S191F variant affects the chemical nature of amino acids from hydrophilic to hydrophobic, it potentially may affect the enzymatic properties of PPAD.

The S203P variant, in turn, co-occurs with previously described seven-nucleotide substitution responsible for a three amino acid G231N, E232T, N235D polymorphism. In analogy to the G231N, E232T, N235D variant, S203P was also produced by *P. gingivalis* strains derived from moderate/advanced and advanced PD. Although the latter is not located close to the active site of PPAD, its co-occurrence with the G231N, E232T, N235D variant may affect bacterial virulence due to the superimposed effect on enzymatic activity and citrullination.

The results indicated that more than half of clinical isolates, i.e., 42/58, harbored point mutations in *ppad* when compared to the *P. gingivalis* reference (ATCC 33277) strain. Altogether, 10 missense mutations were identified in the PD group, with the frequency between 1.72% and 20.69%. Previous studies also reported that clinical isolates of *P. gingivalis* obtained from healthy donors and severe PD donors showed differences in their virulence [35,36], which may be related to the occurrence of specific mutations in the bacterial genes encoding virulence factors, particularly hemagglutinin and fimbriae genes. Moreover, we demonstrated that missense mutations may occur in all stages of PD, however, different mutations have been identified in moderate (M77V, Q373K) and advanced (T275P, P536L) PD independently of their location, e.g., beyond the active center. Interestingly, the co-existence of polymorphic variants, i.e., S191F; G231N, E232T, N235D; S203P and N291D with certain missense mutations, was observed only in advanced PD. Thus, the substitution of methionine with valine in the M77V mutation may alter the properties of the protein, as methionine contains sulfur, which can participate in specific interactions or contribute to structural features. The absence of sulfur in valine may affect interactions with ligands that rely on the presence of methionine. Furthermore, valine is smaller and more rigid, which could affect the local folding of the protein. If methionine is located within the hydrophobic core of the enzyme, its replacement may lead to minor changes in protein stability [33,34]. In the case of the Q373K mutation, glutamine can stabilize substrates or co-enzymes through hydrogen bonding. Its replacement with lysine, which is positively charged, may introduce new electrostatic interactions that could disrupt substrate binding. Lysine has a longer and more flexible side chain than glutamine, potentially altering local protein folding or interactions between different parts of the enzyme [33,34]. Consequently, these mutations, particularly in conjunction with others, may influence the virulence of *P. gingivalis*, predisposing to advanced PD. Similarly, missense mutations associated with advanced PD, such as T275P, may disrupt local secondary structure and reduce protein stability, particularly if threonine is involved in a network of hydrogen bonds. In addition, proline may hinder proper protein folding in this region [33,34]. As for the P536L mutation, substitution of proline by leucine may increase local flexibility and impact the structural stability of the enzyme, potentially impairing its function in a critical region [33,34]. For better understanding of the mechanisms by which these mutations affect PPAD function, structural analysis, such as, molecular modeling, assessments of protein dynamics, or enzymatic assays need to be addressed.

As expected, the most numerous group of changes in the *ppad* gene was represented by synonymous variants, i.e., 35 nucleotide substitutions in PD and 22 in healthy donors, which would suggest their insignificance in the pathogenesis of PD. Otherwise, specific synonymous variants occurred at different stages of PD, i.e., moderate and advanced. Additionally, specific synonymous variants co-existed with the G231N, E232T, N235D variant in PD. Although synonymous variants do not affect the structure of the encoded protein, their presence in the *ppad* gene altogether with the super-active, G231N, E232T, N235D variant, and possibly other changes, should not be neglected in future analyses.

Amongst the genetic changes of the *ppad* gene discussed herein, the most interesting candidate for further virulence tests was the G231N, E232T, N235D variant. It is justified by the effect of this mutation on the primary structure of the enzyme and its presence in the vicinity of the catalytic triad, which corroborates with an increase in enzymatic activity compared to activity reported by wt ATCC 33277 *P. gingivalis*. Moreover, it was detected in ~25% of *P. gingivalis* strains isolated from PD patients and was associated with worse clinical parameters of periodontal tissues in advanced PD [30].

The virulence of the G231N, E232T, N235D variant was evaluated indirectly via the expression analysis of pro-inflammatory cytokines and genes involved in the synthesis of PGE_2_, employing an in vitro model of gingival fibroblasts infected with clinical strains of *P. gingivalis* harbouring or w/o analyzed polymorphic variant from donors with advanced PD, *P. gingivalis* ATCC 33277 with an inserted polymorphism (called ATCC T2), a reference wt-*P. gingivalis* strain (ATCC 33277), and a couple of PPAD controls.

We showed a significant induction of immune response (significant upregulation of *TNF-α* and *IL-6*) by PHGFs infected with each *P. gingivalis* strain harbouring the G231N, E232T, N235D variant in comparison with expression of both genes due to infection with *wt-P. gingivalis*, a clinical strain from advanced PD w/o this polymorphism, or the C351A strain producing inactive PPAD.

The effect of the analyzed polymorphic variant on the PGE_2_-dependent pathway was particularly observed in the case of *COX-2* and *COX-1*. The most elevated expression level of *COX-2* was found as a result of PHGF infection with ATCC T2 and *P. gingivalis* harbouring the polymorphic variant from a donor with advanced PD, while a significant increase in *COX-1* was noted after infection with ATCC T2. Notably, these effects were not observed following cell infection with the *P. gingivalis* strain w/o analyzed variant that was obtained from advanced PD.

The expression of *mPGES-1* was elevated when infected with each *P. gingivalis* strain, excluding the C351A mutant. However, we did not find any differences among cell responses due to infection with strains expressing the G231N, E232T, N235D variant, without this variant and wt ATCC 33277 *P. gingivalis.*

The PPAD is produced by *P. gingivalis* strains and is not derived from host cells. This is consistent with findings from studies indicating that bacterial PPAD plays a role in modulating host immune responses. Host cells, however, produce their own peptidylargine deiminases (PADs), including PAD1-4 and PAD6 [20]. In this study, the activity of human PAD enzymes was not analyzed, however, responses of uninfected cells or cells infected with the ATCC 33277 control strain, which produces a catalytically inactive form of PPAD (C351A), suggest that observed inflammatory effects were more likely caused by bacterial PPAD than any host PAD activity.

Summarizing, many scientific reports highlight PPAD as an attractive virulence factor of *P. gingivalis*. It is particularly elicited by its role in specific protein modification, i.e., citrullination. The modification of free arginine, which is associated with ammonia production, altogether provides energy during anaerobic growth and enables the survival of *P. gingivalis* in periodontal pockets [13,16,17]. The presence of PPAD in the outer membrane of *P. gingivalis* cells enables it to modify the cell-envelope proteins of *P. gingivalis*, as well as host proteins [20]. Citrullination catalyzed by PPAD decreases the charge of modified proteins, thus, it may affect protein stability, sensitivity to proteolysis, and biological activity. Several examples highlight the effects of PPAD citrullination on the function of crucial cytokines and pathways activated in PD, as exemplified by the citrullination of the carboxy-terminal arginine of EGF by PPAD, which may contribute to tissue damage and delayed healing of periodontium in PD [21] or significant upregulation of the PGE_2_-dependent pathway and effective adhesion to and invasion of fibroblasts by *P. gingivalis* producing active PPAD [22]. Moreover, the citrullination of bacterial and host proteins by PPAD is considered a molecular mechanism for generating antigens that initiate and/or enhance the autoimmune response in RA [20,24,26,28]. All the above examples highlight the crucial role of PPAD activity in the pathogenesis of both, PD and RA. Accordingly, the current work was focused on analyses of *ppad* gene sequences from PD donors and the classification and description of all identified *ppad* variants with dependence on the type of mutation, location, occurrence in population, clinical stage of PD, and comparison with *ppad* sequences from *P. gingivalis* strains obtained from healthy donors. Further research into this topic is required for better characterization of the nature of PPAD in the context of its use as an attractive candidate for therapies of PD and associated diseases.

Taking into consideration the limitations of the current study, this paper reports the screening data of the *ppad* gene sequence from clinical vs. reference *P. gingivalis* strains while employing a relatively low number of samples from various stages of PD, which does not enable specification of broad population-based observations. Therefore, further analyses using *P. gingivalis* strains from more numerous groups of PD and control donors are required. Also, all analyses against proximity to the active site were carried out based on the secondary structure of the enzyme, and distance-based modeling of the crystallographic structure was not performed.

## 4. Materials and Methods

### 4.1. Study Participants

This study was conducted in accordance with the Declaration of Helsinki and was approved by the Bioethics Committee of the Jagiellonian University, Medical College, in Krakow, Poland (KBET/310/B/2012; 1072.6120.156.2019). Prior to the study, all participants were instructed on the inclusion and exclusion criteria, and written informed consent was obtained from all donors. The study group consisted of 138 donors diagnosed with PD (aged 22–80 years; 96 females, 42 males), based on the criteria described below. The exclusion criteria included the following: diagnosis of a medical condition, which required pre-medication/pre-treatment prior to dental visits and procedures; at least five decayed and previously untreated teeth at screening (cavities); diagnosis of other diseases of the hard or soft oral tissues; use of any antibiotics or antimicrobial drugs 30 days prior to the qualifying visit; a history of systemic diseases, such as RA, aspiration pneumonia, diabetes mellitus, atherosclerosis, or other uncharacterized systemic diseases; pregnancy and lactation; tobacco smoking; and conditions with impaired tolerance of the immune system, e.g., AIDS or HIV carriers, immunosuppressant intake, radiotherapy, or chemotherapy. The control group comprised 69 donors in good general health without inflammatory diseases of oral cavity and periodontium, including PD and other systemic diseases (aged 18–46 years; 50 females, 19 males).

### 4.2. Periodontal Examination and Sample Collection

Clinical examination of PD and control donors and sample collection were carried out at the Department of Periodontology, Preventive Dentistry and Oral Pathology; and the Department of Oral Surgery, Medical College, Jagiellonian University, in Krakow, Poland. PD was diagnosed on the base of dental and medical history, radiographs, and periodontal examination (probing pocket depth—PPD and clinical attachment level—CAL measurements) of each PD and control donor. Study participants were asked to refrain from eating and drinking from 11:00 p.m. of the night preceding sample collection and to brush their teeth in the evening before the visit but not on the morning of collection. Pooled samples of gingival crevicular fluid (GCF) were collected from five gingival pockets within areas of active inflammation per each PD donor and from five random gingival crevices per each control donor.

### 4.3. P. gingivalis Culturing from GCF Samples

GCF samples collected from PD and control donors were diluted with sterile phosphate-buffered saline (PBS) (1:2) and grown on blood agar plates (BHI, brain heart infusion supplemented with 5% defibrinated sheep blood, 5 g·L^−1^ of yeast extract, 500 μg·mL^−1^ of L-cysteine, 10 µg·mL^−1^ of hemin, 0.5 µg·mL^−1^ of menadione) in an anaerobic chamber (85% N2, 10% CO2, and 5% H2) for 10–14 days. Then, black pigmented colonies were streaked on fresh blood agar plates, and streaking was repeated every 7 days until black-pigment-producing bacteria, possibly *P. gingivalis,* were obtained and preserved in BHI with 20% glycerol at −80 °C. In line with previous reports [37,38], one *P. gingivalis* strain per individual (PD, control) donor was used for further analyses.

### 4.4. P. gingivalis Genomic DNA Purification

Black-pigmented colonies and possible *P. gingivalis* clinical strains were cultured on blood agar plates for 7–10 days, as previously described. Subsequently, bacterial cells were inoculated into enriched BHI broth for overnight culture. For the isolation of bacterial genomic DNA, 2 × 10^9^ bacterial cells were collected (considering that an optical density of 1.0 at 600 nm corresponds to 1 × 10^9^ bacterial cells per 1 mL). Genomic DNA was purified using GeneJET Genomic DNA Purification Kit (Thermo Scientific, Waltham, MA, USA) according to the manufacturer’s instructions. In brief, bacterial samples were centrifuged for 10 min at 5000 rcf and resuspended in digestion buffer (20mM Tris-HCl, pH 8.0; 2mM EDTA; 1.2% Triton X-100; 20 mg·mL^−1^ of lysozyme) for 30 min incubation at 37 °C. Subsequently, lysis buffer and proteinase K were added to samples and incubated for 30 min at 56 °C while shaking, followed by 10 min digestion with RNase at RT. Obtained lysates were applied to the columns and rinsed. The obtained DNA was suspended in 50 µL of ultra-sterile nuclease-free water and preserved for further analysis.

### 4.5. Verification of P. gingivalis Species Genotype

DNA isolated from black-pigment-producing bacteria, indicating *P. gingivalis* clinical strains, was used in PCR reaction to verify species genotype. For this purpose, two reactions were performed, one targeting *P. gingivalis 16S rRNA* gene and second for the full *ppad* gene (Table 8). Reactions were performed using DreamTaq Green PCR Master Mix (Thermo Scientific, Waltham, MA, USA) with 2 µL of template DNA. The following reaction profiles were used: (i) 3 min at 95 °C, (ii) 40 cycles of 30 s at 95 °C, (iii) 30 s at 60 °C, (iv) 72 °C for 60 s (*16S rRNA*)/2 min (*ppad*), and v) final extension for 5 min at 72 °C. Amplification products of the reactions were separated in 1.5% agarose gel stained with ethidium bromide in TAE buffer (40 mM Tris, 20mM acetic acid, 1 mm of EDTA). The presence of specific products of both PCR reactions was required to confirm *P. gingivalis* species.

### 4.6. Cloning and Sequencing of ppad

The whole *P. gingivalis ppad* gene sequence was amplified using Phusion polymerase in HF buffer (Thermo Scientific, Waltham, MA, USA), full *ppad* gene primers (Table 8), and 200 ng of genomic DNA as a template. The reaction profile used was as follows: (i) 30 s at 98 °C, (ii) 40 cycles of 10 s at 98 °C, 15 s at 61 °C, 1 min at 72 °C, and (iii) final extension for 5 min at 72 °C. PCR products were then separated in 1% agarose gel and purified with GeneJET Gel Extraction Kit (Thermo Scientific, Waltham, MA, USA). The purified DNA was suspended in 20 µL of ultra-sterile nuclease-free water and mixed with an identical volume of DreamTaq Green PCR Master Mix (Thermo Scientific, Waltham, MA, USA), followed by incubation at 72 °C for 10 min to place free adenines on the 3′ ends of the DNA. Next, modified products were purified using GeneJET PCR Purification Kit (Thermo Scientific, Waltham, MA, USA). Finally, *ppad* sequences were cloned into pTZ57R/T plasmid using InsTAclone PCR Cloning Kit (Thermo Scientific, Waltham, MA, USA) and transformed into *E. coli* DH5α strain. In brief, the competent *E. coli* strain DH5α was prepared using calcium chloride. For this purpose, 10 mL of LB (Lysogeny broth) medium was inoculated with a single colony of DH5α strain and incubated overnight at 37 °C with shaking. In the next step, 5 mL of culture was used to inoculate 95 mL of fresh LB medium and incubated at 37 °C with shaking. The culture was then chilled on ice for 15 min and also centrifuged for 15 min at 4500 rcf, 4 °C. The bacterial cells pellet was suspended in 50 mL of ice-cold 50 mM calcium chloride solution and incubated on ice for 15 min, then centrifuged under the same conditions as before. Subsequently, the bacterial pellet was suspended in 1 mL of cold 50 mM calcium chloride with 10% glycerol and stored at −80 °C until transformation. During transformation, 5 µL of the ligation mixture was added to 100 μL of bacterial cell solution and incubated for 30 min on ice. The bacteria were shocked at 42 °C water bath for 30 s, transferred to the ice for 2 min, and 950 µL of LB was added. Samples were incubated at 37 °C for 20 min with shaking. Following transformation, 100 µL of the sample was plated on LB broth with 50 µg·mL^−1^ of ampicillin and incubated overnight with shaking at 37 °C. Plasmids in triplicate were purified from cultures with GeneJET Plasmid Miniprep Kit (Thermo Scientific, Waltham, MA, USA). For this purpose, the bacterial culture was centrifuged for 2 min at 4500 rcf, and the pellet was resuspended in 250 µL of RNase A suspension buffer. Cell lysis was performed by adding 250 µL of lysis buffer. The contents were mixed thoroughly by inverting the tube up and down several times. Then, 350 µL of neutralizing buffer was added, and the contents were mixed again. The lysate was centrifuged for 5 min at 13,000 rcf. The supernatant was then transferred to the column, which was rinsed according to the manufacturer’s instructions. Plasmid DNA was eluted in 50 µL of ultra-sterile nuclease-free water. The presence of *ppad* inserts in plasmids was confirmed by PCR with specific primers used. The *ppad* gene was sequenced by Genomed S.A. (Warsaw, Poland) using standard—M13fwd and M13rev primers. The sequences were obtained from three sequencing replicates.

### 4.7. The ppad Gene Sequences Analysis

The *ppad* gene sequences from clinical *P. gingivalis* strains, which were generated based on the consensus sequence from 3 sequencing reads, were analyzed vs. reference wild-type *P. gingivalis* ATCC 33277 strain using NCBI, BLAST, UNIPROT PROTEIN DATABASE, and Expasy.

Additionally, the comparative analysis of the *ppad* gene sequences used nucleotide sequences deposited in the public NCBI database. In the first step, sequences were obtained by searching the nucleotide database using appropriate keywords such as “*ppad gene*”, “peptidylarginine deiminase *P. gingivalis*”, and filtering restrictions to target organisms—“*P. gingivalis*”. The collected sequences were then verified for completeness and quality and compared with the available data to ensure that they represented different *P. gingivalis* strains and isolates. In the next steps, the vs. reference wild-type *P. gingivalis* ATCC 33277 strain analysis was performed using the BLAST program. These results were then compared with the sequences of clinical strains obtained in this study, which allowed for the identification of similarities and differences at the nucleotide level.

Data were grouped based on identified nucleotide substitutions and analyzed using Microsoft Office package. Accession numbers of sequences used in this study are listed in Supplementary Tables S1 and S2.

### 4.8. Gingival Cell Culture

PHGFs from two PD donors used in the study were obtained for the purposes of previous study (KBET/310/B/2012) and deposited in the Cell Bank of the Department of Molecular Biology and Genetics, Faculty of Medical Sciences in Katowice, Medical University of Silesia, Katowice, Poland. The isolation procedure of gingival cells was reported elsewhere [22,39]. Cells were cultured in DMEM (Dulbecco’s Modified Eagle’s Medium), High Glucose, supplemented with 10% FBS and antibiotics (penicillin/streptomycin 50 U/mL) at 37 °C in a humidified atmosphere containing 5% CO_2_ until 95% confluency was reached, and then prepared for infection experiments. For this purpose, the culture medium was removed, and the cells were detached from the culture vessel using 3 mL of trypsin solution with EDTA (ethylenediaminetetraacetic acid) for about 5 min at 37 °C. Cell suspension was centrifuged (300 rcf, 10 min, 4 °C), 3 mL of culture medium was added, and the suspension was diluted 10-fold in trypan blue solution before being transferred to an automated cell counter (TC20 Automated Cell Counter, BioRad, San Francisco, CA, USA). Cells were seeded into 24-well plates at a density of 250,000 cells/well for infection experiments. Approximately 20 h after passaging, the culture medium was removed, and the cells were washed three times with PBS without magnesium and calcium ions. The medium was then changed to DMEM with 2% FBS without antibiotics.

### 4.9. Culture and Preparation of P. gingivalis Strains for Infection

*P. gingivalis* bacterial cultures were initiated in 10 mL of BHI medium by transferring bacteria from a culture plate to the medium and incubating for 20 h at 37 °C in an anaerobic atmosphere. The optical density at OD_600_ of the culture was then measured and diluted to an optical density of OD_600_ = 0.1 in fresh culture medium to a final volume of 15 mL, followed by incubation for 20 h in an anaerobic atmosphere at 37 °C. The bacteria were then centrifuged at 4500 rcf for 10 min at 4 °C and resuspended in PBS. The centrifugation and washing with PBS were repeated three times. The resulting bacterial pellet was resuspended in 5 mL of PBS, the optical density at OD_600_ was measured, and a bacterial suspension in PBS with OD_600_ = 1 was prepared. Infection of cells with the reference strain *P. gingivalis* ATCC 33277, selected clinical strains, and control laboratory mutants was conducted at a multiplicity of infection (MOI) of 1:100 for 24 h in DMEM with 2% FBS. After infection, the medium was removed, and the wells containing cells were washed three times with PBS solution.

### 4.10. RNA Isolation and Preparation

For RNA isolation, 0.5 mL of TriReagent was added to each well, followed by pipetting several times. The obtained cell lysates were used for RNA isolation. RNA isolation was performed according to the manufacturer’s instructions. Briefly, 100 μL of chloroform was added into 0.5 mL of lysate, the mixture was vortexed for 10 s, shaken for 10 s, and then left for 5 min at room temperature before centrifugation at 12,000 rcf for 15 min at 4 °C. After centrifugation, the upper aqueous phase was transferred to a new tube containing 250 μL of isopropanol, mixed, and incubated for 5 min at room temperature, then centrifuged at 12,000 rcf for 15 min at 4 °C. After centrifugation, the supernatant was removed, and the RNA pellet was washed twice with 200 μL of cold 70% ethanol and centrifuged at 12,000 rcf for 10 min at 4 °C. The ethanol was then removed, and the RNA pellet was dried and resuspended in 20 μL of nuclease-free water. Each sample was treated with DNase by adding a mixture containing 5 μL of 10× concentrated buffer for Turbo DNase (Thermo Fisher, Waltham, MA, USA), 2.5 μL of nuclease-free water, and 1 μL of Turbo DNase. Samples were incubated for 30 min at 37 °C, 0.5 mL of TriReagent was added, and the entire isolation procedure was repeated. The concentration and purity of the obtained RNA were measured using NanoDrop (ThermoFisher, Waltham, MA, USA).

### 4.11. Reverse Transcription Reaction

The reverse transcription reaction was performed by suspending 400 ng of total RNA in nuclease-free water to a volume of 10 μL, followed by the addition of 2 μL of 10× concentrated random hexamers. Samples were incubated for 10 min at 70 °C, cooled to 25 °C, and transferred to ice. Then, 8 μL of a mixture containing 4.2 μL of nuclease-free water, 2 μL of 10× concentrated buffer for reverse transcriptase, 0.8 μL of 10 mM dNTP mix, and 1 μL of reverse transcriptase solution was added to the samples. The reverse transcription reaction was carried out for 2 h at 37 °C, followed by heating the samples at 85 °C for 5 min. The obtained cDNA was stored at −20 °C.

### 4.12. Gene Expression Analysis

The relative gene expression of *TNF-α*, *IL-6*, *COX-1*, *COX-2*, and *mPGES-1* for assessment of inflammation after infection was quantified using a quantitative real-time PCR (qRT-PCR) with 2 × SYBR Green Master Mix (Sigma-Aldrich, St. Louis, MO, USA). Standardized primer sets and amplification reaction conditions were used for each gene. The sequences of the primers (Table 8) were obtained commercially (Genomed S.A., Warsaw, Poland). The *β-actin* gene was used as a reference gene. The control was served to uninfected cells.

### 4.13. Statistical Analyses

Statistical tests for relative gene expression results were performed using one-way ANOVA and Tukey’s post hoc multiple comparisons test for the testing of differences among all data groups. The data are presented as the means ± SD and were considered statistically significant at *p* < 0.05. The data were analyzed using GraphPad Prism 10 Software.

## 5. Conclusions

Overall, 4 polymorphic variants, 10 missense mutations, and 35 synonymous variants were identified in the *ppad* gene from *P. gingivalis* strains at various stages of PD, indicating significant heterogeneity of this gene sequence. Amongst all identified changes in the *ppad* gene, a seven-nucleotide substitution resulting in three amino acid changes (G231N, E232T, N235D) in the primary structure of the PPAD was found in ~25% of *P. gingivalis* strains isolated from PD patients. This mutation is located near the catalytic triad, in close proximity to His236, and is associated with a two-fold increase in enzymatic activity compared to the reference *P. gingivalis* strain ATCC 33277. Moreover, in vitro experiments revealed that this polymorphic variant upregulated the expression of several key inflammatory mediators. These results, combined with previous observations linking this variant to worse clinical indices of disease severity in advanced PD, suggest its unambiguous impact on the virulence of *P. gingivalis*.

Taking together, our results open a path for searching for novel targets for supportive therapy of PD, indicating the G231N, E232T, N235D polymorphic variant of the *ppad* gene as a potential candidate. Onward studies are required for its further validation, including other mutations identified in *ppad* discussed in the current study.

## Figures and Tables

**Table 1:** Clinical characteristics of study and control groups.

PD DONOR GROUP (N = 58)				
Classification of PD	Number of Donors	Range of Age	Gender	
			Females	Males
mild	2	44–56	2	0
moderate	15	33–73	9	6
moderate/advanced	4	49–69	3	1
advanced	37	32–80	25	12
**CONTROL GROUP (N = 20)**				
Healthy periodontium	20	18–37	18	2

**Table 2:** Summarized changes of the PPAD in the PD and control groups.

Total Changes in PPAD	PD Donors (N = 58)		Control Group (N = 20)	
	N	%	N	%
Total polymorphic variants	4	8.16%	0	0.00%
Total missense mutations	10	20.41%	0	0.00%
Total synonymous variants	35	71.43%	22	100.00%

**Table 3:** Summarized changes of the PPAD according to the clinical condition of PD.

TOTAL PD DONORS [N = 58]			
MILD PD [N = 2]			
Total changes of PPAD	Total polymorphic variants [%]	Total missense mutations [%]	Total synonymous variants [%]
12	2 [16.67%]	1 [8.33%]	9 [75.00%]
MODERATE PD [N = 15]			
Total changes of PPAD	Total polymorphic variants [%]	Total missense mutations [%]	Total synonymous variants [%]
40	3 [7.50%]	7 [17.50%]	30 [75.00%]
MODERATE/ADVANCED PD [N = 4]			
Total changes of PPAD	Total polymorphic variants [%]	Total missense mutations [%]	Total synonymous variants [%]
19	4 [21.05%]	4 [21.05%]	11 [57.90%]
ADVANCED PD [N = 37]			
Total changes of PPAD	Total polymorphic variants [%]	Total missense mutations [%]	Total synonymous variants [%]
49	4 [8.16%]	9 [18.37%]	36 [73.47%]

**Table 4:** Summarized presentation of polymorphic variants of the PPAD in the PD group.

PD DONORS [N = 58]											
Change of Nucleotide Sequence	Nucleotide Site	Base Site	Codon of WT ATCC 33277	Codon of Clinical Strains	Amino Acid of WT ATCC 33277	Amino Acid of WT ATCC 33277	Amino Acid Clinical Strains of Clinical Strains	Chemical Nature of the Amino Acid of Clinical Strains	Variant	N	%
C→T	572	191	TCC	TTC	Serine	Hydrophilic, neutral (hydroxy amino acid)	Phenylalanine	Hydrophobic	S191F	22	37.93%
T→C	607	203	TCC	CCC	Serine	Hydrophilic neutral (hydroxy amino acid)	Phenylalanine	Hydrophobic	S203P	14	24.14%
* G→A	691	231	GGC	AAT	Glycine	Hydrophobic	Asparagine	Hydrophilic, neutral, aspartic acid amide	G231N	13	22.41%
* G→A	692	231	GGC	AAT	Glycine	Hydrophobic	Asparagine	Hydrophilic, neutral, aspartic acid amide	G231N	13	22.41%
* C→T	693	231	GGC	AAT	Glycine	Hydrophobic	Asparagine	Hydrophilic, neutral, aspartic acid amide	G231N	13	22.41%
* G→A	694	232	GAA	ACT	Glutamic acid	Hydrophilic acidic	Threonine	Hydrophilic, neutral	E232T	13	22.41%
* A→C	695	232	GAA	ACT	Glutamic acid	Hydrophilic acidic	Threonine	Hydrophilic, neutral	E232T	13	22.41%
* A→T	696	232	GAA	ACT	Glutamic acid	Hydrophilic acidic	Threonine	Hydrophilic, neutral	E232T	13	22.41%
* A→G	703	235	AAC	GAC	Asparagine	Hydrophilic neutral, aspartic acid amide	Aspartic acid	Hydrophilic, acidic	N235D	13	22.41%
* A→G	871	291	AAT	GAT	Asparagine	Hydrophilic, neutral, aspartic acid amide	Aspartic acid	Hydrophilic, acidic	N291D	24	41.38%

* close proximity to the active site.

**Table 5:** Summarized presentation of missense mutations of the PPAD in the PD group.

PD DONORS [N = 58]											
Change of Nucleotide Sequence	Nucleotide Site	Base Site	Codon of WT ATCC 33277	Codon of Clinical Strains	Amino Acid of WT ATCC 33277	Amino Acid of WT ATCC 33277	Amino Acid Clinical Strains of Clinical Strains	Chemical Nature of the Amino Acid of Clinical Strains	Variant	N	%
C→T	74	25	ACG	ATG	Threonine	Hydrophilic, neutral (hydroxy amino acid)	Methionine	Hydrophobic, neutral	T25M	4	6.90%
A→G	229	77	ATG	GTG	Methionine	Hydrophobic, neutral	Valine	Hydrophobic, neutral	M77V	2	3.45%
A→C	823	275	ACC	CCC	Threonine	Hydrophilic, neutral (hydroxy amino acid)	Proline	Hydrophobic, neutral	T275P	1	1.72%
C→A	1117	373	CAG	AAG	Glutamine	Hydrophilic, neutral	Lysine	Hydrophilic, basic	Q373K	5	8.62%
G→A	1168	390	GCT	ACT	Alanine	Hydrophobic, neutral	Threonine	Hydrophilic, neutral (hydroxy amino acid)	A390T	5	8.62%
C→T	1262	421	ACT	ATT	Threonine	Hydrophilic, neutral (hydroxy amino acid)	Isoleucine	Hydrophobic, neutral	T421I	5	8.62%
C→T	1544	515	GCA	GTA	Alanine	Hydrophobic, neutral	Valine	Hydrophobic, neutral	A515V	12	20.69%
A→T	1556	519	GAA	GTA	Glutamic acid	Hydrophilic, acidic	Valine	Hydrophobic, neutral	E519V	4	6.90%
A→G	1582	528	AGT	GGT	Serine	Hydrophilic, neutral (hydroxy amino acid)	Glycine	Hydrophobic	S528G	12	20.69%
C→T	1607	536	CCG	CTG	Proline	Hydrophobic, neutral	Leucine	Hydrophobic, neutral	P536L	1	1.72%

**Table 6:** Summarized presentation of synonymous variants of the PPAD in the PD and control groups.

PD DONORS [N = 58]								
Changes of Nucleotide Sequence	Nucleotide Site	Codon	Base Side	Amino Acid	Chemical Nature of the Amino Acid	Variant	N	%
G→A	144	ACG→ACA	48	Threonine	Hydrophilic, neutral (hydroxy amino acid)	T48T	12	20.69%
C→T	213	TAC→TAT	71	Tyrosine	Hydrophilic, neutral	Y71Y	3	5.17%
C→T	258	AAC→AAT	86	Asparagine	Hydrophilic, neutral, aspartic acid amide	N86N	3	5.17%
G→A	363	GCG→GCA	121	Alanine	Hydrophobic, neutral	A121A	5	8.62%
* C→T	378	TAC→TAT	126	Tyrosine	Hydrophilic, neutral	Y126Y	2	3.45%
* C→T	405	TTC→TTT	135	Phenylalanine	Hydrophobic	F135F	7	12.07%
C→T	546	GGC→GGT	182	Glycine	Hydrophobic	G182G	14	24.14%
C→T	549	AAC→AAT	183	Asparagine	Hydrophilic, neutral, aspartic acid amide	N183N	2	3.45%
G→T	600	ACG→ACT	200	Threonine	Hydrophilic, neutral (hydroxy amino acid)	T200T	29	50.00%
T→C	618	TCT→TCC	206	Serine	Hydrophilic, neutral (hydroxy amino acid)	S206S	7	12.07%
* A→G	735	GCA→GCG	245	Alanine	Hydrophobic, neutral	A245A	26	44.83%
* C→T	741	AAC→AAT	247	Asparagine	Hydrophilic, neutral, aspartic acid amide	N247N	3	5.17%
G→A	762	GTG→GTA	254	Valine	Hydrophobic, neutral	V254V	23	39.65%
C→T	789	GCC→GCT	263	Alanine	Hydrophobic, neutral	A263A	25	43.10%
C→T	790	CTG→TTG	264	Leucine	Hydrophobic, neutral	L264L	1	1.72%
A→G	852	GTA→GTG	284	Valine	Hydrophobic, neutral	V284V	33	56.90%
* G→A	912	AGG→AGA	304	Arginine	Hydrophilic, basic	R304R	2	3.45%
C→T	927	GTC→GTT	309	Valine	Hydrophobic, neutral	V309V	5	8.62%
C→T	948	GAC→GAT	316	Aspartic acid	Hydrophilic, acidic	D316D	3	5.17%
G→A	960	CTG→CTA	320	Leucine	Hydrophobic, neutral	L320L	7	12.07%
C→T	963	AAC→AAT	321	Asparagine	Hydrophilic, neutral, aspartic acid amide	N321N	21	36.21%
G→A	975	ACG→ACA	325	Threonine	Hydrophilic, neutral (hydroxy amino acid)	T325T	2	3.45%
T→C	987	GGT→GGC	329	Glycine	Hydrophobic	G329G	4	6.90%
* A→C	1035	GGA→GGC	345	Glycine	Hydrophobic	G345G	11	18.96%
* C→T	1080	GGC→GGT	360	Glycine	Hydrophobic	G360G	2	3.45%
A→G	1146	GCA→GCG	382	Alanine	Hydrophobic, neutral	A382A	3	5.17%
T→C	1170	GCC→GCC	390	Alanine	Hydrophobic, neutral	A390A	3	5.17%
A→G	1299	GTA→GTG	433	Valine	Hydrophobic, neutral	V433V	7	12.07%
T→C	1371	CCT→CCC	457	Proline	Hydrophobic, neutral	P457P	5	8.62%
T→A	1422	GCT→GCA	474	Alanine	Hydrophobic, neutral	A474A	4	6.90%
T→C	1437	CGT→CGC	479	Arginine	Hydrophilic, basic	R479R	13	22.41%
T→C	1491	ATT→ATC	497	Isoleucine	Hydrophobic, neutral	I497I	2	3.45%
G→A	1593	GTG→GTA	531	Valine	Hydrophobic, neutral	V531V	5	8.62%
C→T	1611	GCC→GGT	537	Glycine	Hydrophobic	G537G	5	8.62%
C→T	1621	CTG→TTG	541	Leucine	Hydrophobic, neutral	L541L	16	27.59%
**CONTROL GROUP [N = 20]**								
**Change of nucleotide sequence**	**Nucleotide site**	**Codon**	**Amino acid site**	**Amino acid**	**Chemical nature** **of the amino acid**	**Variant**	**N**	**%**
C→T	213	TAC→TAT	71	Tyrosine	Hydrophilic, neutral	Y71Y	6	30.00%
C→T	258	AAC→AAT	86	Asparagine	Hydrophilic, neutral, aspartic acid amide	N86N	6	30.00%
C→T	549	AAC→AAT	183	Asparagine	Hydrophilic, neutral, aspartic acid amide	N183N	6	30.00%
G→T	600	ACG→ACT	200	Threonine	Hydrophilic, neutral (hydroxy amino acid)	T200T	3	15.00%
* A→G	735	GCA→GCG	245	Alanine	Hydrophobic, neutral	A245A	4	20.00%
G→A	762	GTG→GTA	254	Valine	Hydrophobic, neutral	V254V	4	20.00%
C→T	789	GCC→GCT	263	Alanine	Hydrophobic, neutral	A263A	8	40.00%
A→G	852	GTA→GTG	284	Valine	Hydrophobic, neutral	V284V	6	30.00%
* G→A	912	AGG→AGA	304	Arginine	Hydrophilic, basic	R304R	3	20.00%
C→T	948	GAC→GAT	316	Aspartic acid	Hydrophilic, acidic	D316D	6	30.00%
C→T	963	AAC→AAT	321	Asparagine	Hydrophilic, neutral, aspartic acid amide	N321N	4	20.00%
G→A	975	ACG→ACA	325	Threonine	Hydrophilic, neutral (hydroxy amino acid)	T325T	6	30.00%
T→C	987	GGT→GGC	329	Glycine	Hydrophobic	G329G	7	35.00%
A→C	1035	GGA→GGC	345	Glycine	Hydrophobic	G345G	3	15.00%
* C→T	1080	GGC→GGT	360	Glycine	Hydrophobic	G360G	6	30.00%
A→G	1146	GCA→GCG	382	Alanine	Hydrophobic, neutral	A382A	6	30.00%
T→A	1422	GCT→GCA	474	Alanine	Hydrophobic, neutral	A474A	1	5.00%
T→C	1437	CGT→CGC	479	Arginine	Hydrophilic, basic	R479R	1	5.00%
C→A	1452	GCC→GCA	484	Alanine	Hydrophobic, neutral	A484A	1	5.00%
T→C	1491	ATT→ATC	497	Isoleucine	Hydrophobic, neutral	I497I	6	30.00%
G→A	1593	GTG→GTA	531	Valine	Hydrophobic, neutral	V531V	6	30.00%
C→T	1621	CTG→TTG	541	Leucine	Hydrophobic, neutral	L541L	5	25.00%

* close proximity to the active site.

**Table 7:** The characteristics of changes in close proximity to the active center of PPAD.

Active Site	Variant	Type of Changes in PPAD	Classification of PD
Asp130	Y126Y *	Synonymous variant	moderate/advanced
	F135F *	Synonymous variant	moderate moderate/advanced
His236	A245A	Synonymous variant	mild moderate moderate/advanced advanced
	G231N	Polymorphic variant (PD group)	moderate/advancedadvanced
	E232T		
	N235D		
Asp238	N247N *	Synonymous variant	moderate
Asn297	N291D	Polymorphic variant (PD group)	mild moderate moderate/advanced advanced
Cys351	G345G	Synonymous variant	moderate
	G360G	Synonymous variant	mild moderate advanced

* variants present only in the study group.

**Table 8:** Primer sequences used in the study (synthetized by Genomed SA, Warsaw, Poland).

Primer Name	5′→3′ Sequences
*P. gingivalis 16S rRNA*	FOR: AGGCAGCTTGCCATACTGCG
	REV: ACTGTTAGYAACTACCGATGT
*P. gingivalis ppad*_verification (full *ppad*)	FOR: ATGAAAAAGCTTTTACAGGCTAAAGCCTTG
	REV: TTATTTGAGAATTTTCATTGTCTCACGGATTCC
*P. gingivalis ppad*_cloning	FOR: ATGAAAAAGCTTTTACAGGCTAAAGCCTTG
	REV: TTATTTGAGAATTTTCATTGTCTCACGGATTCC
*P. gingivalis ppad*_seqeuncing (M13)	FOR: GTAAAACGACGGCCAGT
	REV: CAGGAAACAGCTATGAC
pUC_19	FOR: GAGCTCGGTACCCGGGGATC
	REV: GAATTCACTGGCCGTCGTTTTACAACG
*TNF-α* (*Homo sapiens*)	FOR: CCCGAGTGACAAGCCTGTAG
	REV: GATGGCAGAGAGGAGGTTGAC
*IL-6* (*Homo sapiens*)	FOR: ACAGCCACTCACCTCTTCAG
	REV: CCATCTTTTTCAGCCATCTTT
*COX-1* (*Homo sapiens*)	FOR: CAGTTGCCAGATGCCCAGCTC
	REV: GTGCATCAACACAGGCGCCTC
*COX-2* (*Homo sapiens*)	FOR: AGCCCTTCCTCCTGTGCCT
	REV: TCCATTTTTCGTCGAAGGACTAA
*mPGES-1* (*Homo sapiens*)	FOR: CACGCTGCTGGTCATCAAGAT
	REV: TCCTACGGGACTCTGTGCC
*β-actin* (*Homo sapiens*)	FOR: CCACACTGTGCCCATCTACG
	REV: AGGATCTTCATGAGGTAGTCAGTCAG

## Data Availability

The original contributions presented in the study are included in the article. Further inquiries can be directed to the corresponding author.

## Supplementary Materials

The following supporting information can be downloaded at: https://www.mdpi.com/article/10.3390/ijms26041662/s1.
