# Supplementary material for: Polymorphic Variants of Peptidylarginine Deiminase Gene from P. gingivalis—Searching for Targets for Supportive Therapy of Periodontitis
Source: Int J Mol Sci. 2025 Feb 15;26(4):1662. doi: 10.3390/ijms26041662 (PMC11855631; doi:10.3390/ijms26041662)
Supplement: Supplementary file 1 [file ijms-26-01662-s001.zip › ijms-3392258-supplementary.pdf]

Table S1. Accession numbers of GenBank sequences analyzed in this study

Accession numbers of GenBank sequences analyzed in this study	
Strain name	Accession number
P. gingivalis ATCC 33277	PD025930
PD1	PP079918
PD2	PP079919
PD3	PP079920
PD4	PP079921
PD5	PP079922
PD6	PP079923
PD7	PP079924
PD8	PP079925
PD9	PP079926
PD10	PP079927
PD11	PP079928
PD12	PP079929
PD13	PP079930
PD14	PP079931
PD15	PP079932
PD16	PP079933
PD17	PP079934
PD18	PP079935
PD19	PP079936
PD20	PP079937
PD21	PP079938
PD22	PP079939
PD23	PP079940
PD24	PQ605366
PD25	PQ605367
PD26	PQ605368
PD27	PQ605369
PD28	PQ605370
PD29	PQ605371
PD30	PQ605372
PD31	PQ605373
PD32	PQ605374
PD33	PQ605375
PD34	PQ605376
PD35	PQ605377
PD36	PQ605378
PD37	PQ605379
PD38	PQ605380
PD39	PQ605381
PD40	PQ605382
PD41	PQ605383
PD42	PQ605384
PD43	PQ605385
PD44	PQ605386

PD45	PQ605387
PD46	PQ605388
PD47	PQ605389
PD48	PQ605390
PD49	PQ605391
PD50	PQ605392
PD51	PQ605393
PD52	PQ605394
PD53	PQ605395
PD54	PQ605396
PD55	PQ605397
PD56	PQ605398
PD57	PQ605399
PD58	PQ605400
CTRL1	PP079941
CTRL2	PP079942
CTRL3	PP079943
CTRL4	PP079944
CTRL5	PP079945
CTRL6	PP079946
CTRL7	PP079947
CTRL8	PP079948
CTRL9	PP691522
CTRL10	PP691523
CTRL11	PP691524
CTRL12	PP691525
CTRL13	PP691526
CTRL14	PP691527
CTRL15	PP691528
CTRL16	PQ605401
CTRL17	PQ605402
CTRL18	PQ605403
CTRL19	PQ605404
CTRL20	PQ605405

Table S2. Accession numbers of GenBank sequences from databases

Accession numbers of GenBank sequences from databases	
Strain name	Accession number
DATABASE1	KP862656.1
DATABASE2	KP862650.1
DATABASE3	KP862652.1
DATABASE4	KP862654.1
DATABASE5	KP862655.1
DATABASE6	KP862653.1
DATABASE7	KP862651.1
DATABASE8	CP011995.1
DATABASE9	AF153769.1
DATABASE10	AP009380.1

DATABASE11	CP073349.1
DATABASE12	CP073350.1
DATABASE13	CP024601.1
DATABASE14	CP024599.1
DATABASE15	CP024591.1
DATABASE16	CP024598.1
DATABASE17	CP025931.1
DATABASE18	CP024592.1
DATABASE19	CP024595.1
DATABASE20	CP024596.1
DATABASE21	CP024600.1
DATABASE22	CP024594.1
DATABASE23	CP024593.1
DATABASE24	CP024597.1
DATABASE25	CP011996.1
DATABASE26	AWUV01000067.1
DATABASE27	AWUW01000017.1
DATABASE28	FUFF01000006.1
DATABASE29	AWUU01000015.1
DATABASE30	AWUU01000015.1
DATABASE31	ASYN01000031.1
DATABASE32	AWVE01000083.1
DATABASE33	FUFD01000013.1
DATABASE34	AWVD01000025.1
DATABASE35	AWVC01000009.1
DATABASE36	ASYO01000090.1
DATABASE37	ASYP01000102.1
DATABASE38	AJZS01000007.1
DATABASE39	FUGG01000063.1
DATABASE40	LOEL01000042.1
DATABASE41	FUFG01000025.1
DATABASE42	FUFC01000005.1
DATABASE43	ASYM01000109.1
DATABASE44	ASYL01000006.1
DATABASE45	FUFB01000005.1
DATABASE46	FUFE01000062.1
DATABASE47	NSLX01000047.1
DATABASE48	NSLW01000004.1
DATABASE49	NHRU01000002.1
DATABASE50	BCBV01000036.1
DATABASE51	NSLT01000080.1
DATABASE52	NSLV01000014.1
DATABASE53	FUFH01000058.1
DATABASE54	FUFJ01000053.1
DATABASE55	NSLS01000062.1
DATABASE56	NSLU01000028.1
DATABASE57	FUGF01000032.1
DATABASE58	NSLN01000009.1
DATABASE59	SGBA01000041.1
DATABASE60	NSLL01000036.1

Table S3. Polymorphic variants of peptidylaginine deiminase gene sequences from *P. gingivalis* deposited in databases (n=60)

Change of nucleotide sequence	Nucleotide site	Base site	Codon of WT ATCC 33277	Codon of clinical strains	Amino acid of WT ATCC 33277	Amino acid of WT ATCC 33277	Amino acid clinical strains of clinical strains	Chemical nature of the amino acid of clinical strains	Variant	N	%
T→C	607	203	TCC	CCC	Serine	Hydrophilic, neutral (hydroxy amino acid)	Phenylalanine	Hydrophobic	S203P	18	30.00%
*G→A	691	231	GGC	AAT	Glycine	Hydrophobic	Asparagine	Hydrophilic, neutral, aspartic acid amide	G231N	15	25.00%
*G→A	692	231	GGC	AAT	Glycine	Hydrophobic	Asparagine	Hydrophilic, neutral, aspartic acid amide	G231N	15	25.00%
*C→T	693	231	GGC	AAT	Glycine	Hydrophobic	Asparagine	Hydrophilic, neutral, aspartic acid amide	G231N	15	25.00%
*G→A	694	232	GAA	ACT	Glutamic acid	Hydrophilic, acidic	Threonine	Hydrophilic, neutral	E232T	15	25.00%
*A→C	695	232	GAA	ACT	Glutamic acid	Hydrophilic, acidic	Threonine	Hydrophilic, neutral	E232T	15	25.00%
*A→T	696	232	GAA	ACT	Glutamic acid	Hydrophilic, acidic	Threonine	Hydrophilic, neutral	E232T	15	25.00%
*A→G	703	235	AAC	GAC	Asparagine	Hydrophilic, neutral, aspartic acid amide	Aspartic acid	Hydrophilic, acidic	N235D	15	25.00%
*A→G	871	291	AAT	GAT	Asparagine	Hydrophilic, neutral, aspartic acid amide	Aspartic acid	Hydrophilic, acidic	N291D	24	40.00%

* close proximity to the active site.

Table S4. Missense mutations of peptidylaginine deiminase gene sequences from *P. gingivalis* deposited in databases (n=60)

Change of nucleotide sequence	Nucleotide site	Base site	Codon of WT ATCC 33277	Codon of clinical strains	Amino acid of WT ATCC 33277	Amino acid of WT ATCC 33277	Amino acid clinical strains of clinical strains	Chemical nature of the amino acid of clinical strains	Variant	N	%
A→G	79	27	ATG	GTG	Methionine	Hydrophobic, neutral	Valine	Hydrophobic, neutral	M27V	2	3.33%
C→T	155	52	GCA	GTA	Alanine	Hydrophobic, neutral	Valine	Hydrophobic, neutral	A52V	1	1.67%
A→G	229	77	ATG	GTG	Methionine	Hydrophobic, neutral	Valine	Hydrophobic, neutral	M77V	11	18.33%
G→C	280	94	GCG	CAA	Alanine	Hydrophobic, neutral	Glutamin e	Hydrophilic, neutral	A94Q	1	1.67%
C→A	281	94	GCG	CAA	Alanine	Hydrophobic, neutral	Glutamin e	Hydrophilic, neutral	A94Q	1	1.67%
C→A	282	94	GCG	CAA	Alanine	Hydrophobic, neutral	Glutamin e	Hydrophilic, neutral	A94Q	1	1.67%
G→A	285	95	AGT	AAC	Serine	Hydrophilic, neutral (hydroxy amino acid)	Asparagi ne	Hydrophilic, neutral , aspartic acid amide	S95N	1	1.67%
T→C	286	95	AGT	AAC	Serine	Hydrophilic, neutral (hydroxy amino acid)	Asparagi ne	Hydrophilic, neutral , aspartic acid amide	S95N	1	1.67%
G→A	290	97	AGC	AAC	Serine	Hydrophilic, neutral (hydroxy amino acid)	Asparagi ne	Hydrophilic, neutral , aspartic acid amide	S97N	1	1.67%
A→G	298	10 0	AAC	GGC	Asparagine	Hydrophilic, neutral, aspartic acid amide	Glycine	Hydrophobic	N100G	1	1.67%
A→G	299	10 0	AAC	GGC	Asparagine	Hydrophilic, neutral, aspartic acid amide	Glycine	Hydrophobic	N100G	1	1.67%
C→T	302	10 1	ACC	ATC	Threonine	Hydrophilic, neutral (hydroxy amino acid)	Isoleucine	Hydrophobic, neutral	T101I	1	1.67%
C→A	311	10 4	AGC	AAC	Threonine	Hydrophilic, neutral (hydroxy amino acid)	Asparagi ne	Hydrophilic, neutral, aspartic acid amide	T104N	1	1.67%
C→A	313	10 5	CAG	AAG	Glutamine	Hydrophilic, neutral	Lysine	Hydrophilic, basic	Q105K	1	1.67%

G→A	326	109	AGC	AAC	Serine	Hydrophilic, neutral (hydroxy amino acid)	Asparagine	Hydrophilic, neutral, aspartic acid amide	S109N	1	1.67%
*G→A	361	121	GCG	ACT	Alanine	Hydrophobic, neutral	Threonine	Hydrophilic, neutral (hydroxy amino acid)	A121T	1	1.67%
*G→A	363	121	GCG	ACT	Alanine	Hydrophobic, neutral	Threonine	Hydrophilic, neutral (hydroxy amino acid)	A121T	1	1.67%
*A→T	364	122	AAA	TAC	Lysine	Hydrophilic, basic	Tyrosine	Hydrophilic, neutral	K122Y	1	1.67%
*A→C	366	122	AAA	TAC	Lysine	Hydrophilic, basic	Tyrosine	Hydrophilic, neutral	K122Y	1	1.67%
*A→G	394	132	ACC	GCC	Threonine	Hydrophilic, neutral (hydroxy amino acid)	Alanine	Hydrophobic, neutral	T132A	1	1.67%
*G→A	406	136	GCA	ATA	Alanine	Hydrophobic, neutral	Isoleucine	Hydrophobic, neutral	A136I	1	1.67%
*C→T	407	136	GCA	ATA	Alanine	Hydrophobic, neutral	Isoleucine	Hydrophobic, neutral	A136I	1	1.67%
C→T	458	153	CCT	CAT	Proline	Hydrophobic, neutral	Histidine	Hydrophilic, basic	P153H	2	3.33%
A→T	477	159	GAA	GAT	Glutamic acid	Hydrophilic, acidic	Aspartic acid	Hydrophilic, acidic	E159D	1	1.67%
C→T	572	191	TCC	TTC	Serine	Hydrophilic, neutral (hydroxy amino acid)	Phenylalanine	Hydrophobic	S191F	13	20.00%
A→C	823	275	ACC	CCC	Threonine	Hydrophilic, neutral (hydroxy amino acid)	Proline	Hydrophobic, neutral	T275P	3	5.00%
*C→T	881	294	CCG	CTG	Proline	Hydrophobic, neutral	Leucine	Hydrophobic, neutral	P294L	1	1.67%
C→A	958	320	CTG	ATG	Leucine	Hydrophobic, neutral	Methionine	Hydrophobic, neutral	L320M	1	1.67%
G→A	1003	335	GTC	ATC	Valine	Hydrophobic, neutral	Isoleucine	Hydrophobic, neutral	V335I	9	15.00%
*C→T	1070	357	GCG	GTG	Alanine	Hydrophobic, neutral	Valine	Hydrophobic, neutral	A357V	1	1.67%
C→A	1117	373	CAG	AAG	Glutamine	Hydrophilic, neutral	Lysine	Hydrophilic, basic	Q373K	6	10.00%
G→A	1168	390	GCT	ACT	Alanine	Hydrophobic, neutral	Threonine	Hydrophilic, neutral (hydroxy amino acid)	A390T	4	6.67%

G→A	1168	41<br>0	GCT	ACT	Alanine	Hydrophobic, neutral	Threonin<br>e	Hydrophilic, neutral (hydroxy amino acid)	A410T	3	5.00%
G→A	1228	41<br>7	ACA	ATA	Threonine	Hydrophilic, neutral (hydroxy amino acid)	Isoleucine	Hydrophobic, neutral	T417I	1	1.67%
C→T	1262	42<br>1	ACT	ATT	Threonine	Hydrophilic, neutral (hydroxy amino acid)	Isoleucine	Hydrophobic, neutral	T421I	1	1.67%
A→G	1273	42<br>5	ACA	GCA	Threonine	Hydrophilic, neutral (hydroxy amino acid)	Alanine	Hydrophobic, neutral	T425A	3	5.00%
C→T	1523	50<br>8	ACC	ATC	Threonine	Hydrophilic, neutral (hydroxy amino acid)	Isoleucine	Hydrophobic, neutral	T508I	2	3.33%
C→T	1544	51<br>5	GCA	GTA	Alanine	Hydrophobic, neutral	Valine	Hydrophobic, neutral	A515V	6	10.00%
A→G	1582	52<br>8	AGT	GGT	Serine	Hydrophilic, neutral (hydroxy amino acid)	Glycine	Hydrophobic	S528G	3	5.00%
C→T	1607	53<br>6	CCG	CTG	Proline	Hydrophobic, neutral	Leucine	Hydrophobic, neutral	P536L	1	1.67%
G→A	1640	54<br>7	GGA	GAA	Glycine	Hydrophobic	Glutamic acid	Hydrophilic, acidic	G547E	1	1.67%
C→T	1652	55<br>1	ACA	ATA	Threonine	Hydrophilic, neutral (hydroxy amino acid)	Isoleucine	Hydrophobic, neutral	T551I	1	1.67%

* close proximity to the active site.

Table S5. Synonymous variants of peptidylaginine deiminase gene sequences from *P. gingivalis* deposited in databases (n=60)

Changes of nucleotide sequence	Nucleotide site	Codon	Base side	Amino acid	Chemical nature of the amino acid	Variant	N	%
T→A	21	GCT→GCA	7	Alanine	Hydrophobic, neutral	A7A	2	3.33%
G→A	144	ACG→ACA	48	Threonin<br>e	Hydrophilic, neutral (hydroxy amino acid)	T48T	11	18.33%
C→T	213	TAC→TAT	71	Tyrosine	Hydrophilic, neutral	Y71Y	38	63.33%

G→A	249	CTG→CTA	83	Leucine	Hydrophobic, neutral	L83L	1	1.67%
C→T	258	AAC→AAT	86	Asparagine	Hydrophilic, neutral, aspartic acid amide	N86N	30	50.00%
G→A	279	GTG→GTA	93	Valine	Hydrophobic, neutral	V93V	1	1.67%
T→C	306	GTT→GTC	102	Valine	Hydrophobic, neutral	V102V	1	1.67%
A→C	309	ATA→ATC	103	Isoleucine	Hydrophobic, neutral	I103I	1	1.67%
C→T	318	TAC→TAT	106	Tyrosine	Hydrophilic, neutral	Y106Y	1	1.67%
C→G	321	ACC→ACG	107	Threonine	Hydrophilic, neutral (hydroxy amino acid)	T107T	1	1.67%
A→G	324	CAA→CAG	108	Glutamine	Hydrophilic, neutral	Q108Q	1	1.67%
*G→A	363	GCG→ACT	121	Alanine	Hydrophobic, neutral	A121A	2	3.33%
*T→G	369	ACT→ACG	123	Threonine	Hydrophilic, neutral (hydroxy amino acid)	T123T	2	3.33%
*C→T	378	TAC→TAT	126	Tyrosine	Hydrophilic, neutral	Y126Y	2	3.33%
*C→T	405	TTC→TTT	135	Phenylalanine	Hydrophobic	F135F	5	8.33%
T→C	474	GAT→GAC	158	Aspartic acid	Hydrophilic, acidic	D158D	1	1.67%
C→T	546	GGC→GGT	182	Glycine	Hydrophobic	G182G	24	40.00%
C→T	549	AAC→AAT	183	Asparagine	Hydrophilic, neutral, aspartic acid amide	N183N	35	58.33%
G→T	600	ACG→ACT	200	Threonine	Hydrophilic, neutral (hydroxy amino acid)	T200T	13	21.67%
T→C	618	TCT→TCC	206	Serine	Hydrophilic, neutral (hydroxy amino acid)	S206S	19	31.67%
*A→G	735	GCA→GCG	245	Alanine	Hydrophobic, neutral	A245A	21	35.00%
C→T	753	GTG→GTA	251	Isoleucine	Hydrophobic, neutral	I251I	1	1.67%
G→A	762	GTG→GTA	254	Valine	Hydrophobic, neutral	V254V	27	45.00%
T→A	765	CCT→CCA	255	Proline	Hydrophobic, neutral	P255P	1	1.67%
C→T	789	GCC→GCT	263	Alanine	Hydrophobic, neutral	A263A	28	46.67%
A→G	852	GTA→GTG	284	Valine	Hydrophobic, neutral	V284V	31	51.67%
C→T	858	CGC→CGT	286	Arginine	Hydrophilic, basic	R286R	4	6.67%

*G→A	912	AGG→AGA	304	Arginine	Hydrophilic, basic	R304R	3	5.00%
C→T	927	GTC→GTT	309	Valine	Hydrophobic, neutral	V309V	1	1.67%
C→A	942	TCC→TCA	314	Serine	Hydrophilic, neutral (hydroxy amino acid)	S314S	1	1.67%
C→T	948	GAC→GAT	316	Aspartic acid	Hydrophilic, acidic	D316D	50	83.33%
C→T	963	AAC→AAT	321	Asparagine	Hydrophilic, neutral, aspartic acid amide	N321N	14	23.33%
G→A	975	ACG→ACA	325	Threonine	Hydrophilic, neutral (hydroxy amino acid)	T325T	3	5.00%
T→C	987	GGT→GGC	329	Glycine	Hydrophobic	G329G	19	31.67%
T→A	1002	GGT→GGA	334	Glycine	Hydrophobic	G334G	2	3.33%
T→A	1026	CCT→CCA	342	Glycine	Hydrophobic	G342G	4	6.67%
*A→C	1035	GGA→GGC	345	Glycine	Hydrophobic	G345G	23	38.33%
*C→T	1080	GGC→GGT	360	Glycine	Hydrophobic	G360G	7	11.67%
C→T	1108	CTG→TTG	370	Leucine	Hydrophobic, neutral	L370L	3	5.00%
C→T	1125	GGC→GGT	375	Glycine	Hydrophobic	G375G	4	6.67%
A→G	1146	GCA→GCG	382	Alanine	Hydrophobic, neutral	A382A	14	23.33%
T→C	1170	GCC→GCC	390	Alanine	Hydrophobic, neutral	A390A	6	10.00%
C→T	1323	GAC→GAT	441	Aspartic acid	Hydrophilic, acidic	D441D	1	1.67%
T→C	1371	CCT→CCC	457	Proline	Hydrophobic, neutral	P457P	8	13.33%
G→T	1383	ACG→ACT	461	Threonine	Hydrophilic, neutral (hydroxy amino acid)	T461T	1	1.67%
T→A	1422	GCT→GCA	474	Alanine	Hydrophobic, neutral	A474A	17	28.33%
T→C	1437	CGT→CGC	479	Arginine	Hydrophilic, basic	R479R	10	16.67%
C→T	1449	AAC→AAT	483	Asparagine	Hydrophilic, neutral, aspartic acid amide	N483N	1	1.67%
C→A	1452	GCC→GCA	484	Alanine	Hydrophobic, neutral	A484A	10	16.67%
T→C	1491	ATT→ATC	497	Isoleucine	Hydrophobic, neutral	I497I	38	63.33%

G→A	1593	GTG→GTA	531	Valine	Hydrophobic, neutral	V531V	59	98.33%
C→T	1611	GCC→GGT	537	Glycine	Hydrophobic	G537G	4	6.67%
C→T	1621	CTG→TTG	541	Leucine	Hydrophobic, neutral	L541L	34	56.67%

* close proximity to the active site.
